# Knowledge of HIV and/or AIDS and HIV testing services among young men in South Africa

**DOI:** 10.4102/phcfm.v15i1.3796

**Published:** 2023-07-31

**Authors:** Sithembiso M. S. Ndlovu, Andrew Ross, James Ndirangu

**Affiliations:** 1 Department of Family Medicine, Faculty of Health Sciences, University of KwaZulu-Natal, Durban, South Africa; 2 Office of the Dean of Health Sciences, Faculty of Health Sciences, University of the Free State, Bloemfontein, South Africa

**Keywords:** young men, HIV and/or AIDS, HIV testing services (HTS), knowledge, Ladysmith, HIV self-testing (HIVST)

## Abstract

**Background:**

The youth is at a heightened risk of immunodeficiency virus and/or acquired immunodeficiency syndrome (HIV and/or AIDS) infection because of risk-taking behaviour. There remains a gap in understanding young men’s knowledge of HIV and/or AIDS and HIV testing services (HTS) in hard-to-reach communities in South Africa.

**Aim:**

This article aimed to explore young men’s knowledge of HIV and/or AIDS, including HTS in Ladysmith, KwaZulu-Natal (KZN).

**Setting:**

Rural and peri-urban areas around the town of Ladysmith.

**Methods:**

Employing a qualitative descriptive research design, 17 young men aged between 18 and 30 years were purposively and conveniently sampled and interviewed using WhatsApp and landline audio calls to collect their data, which was thematically analysed.

**Results:**

Young men had good knowledge of HIV and/or AIDS but lacked knowledge about HTS and HIV self-testing (HIVST). They obtained their information about HIV and/or AIDS and HTS from various sources and were aware of where to access HTS. They were generally unaware and supportive of HIVST.

**Conclusion:**

Male-targeted HIV and/or AIDS knowledge and testing interventions are needed to encourage and support young men to test for HIV. Human immunodeficiency virus self-testing should be explored as an alternative to clinic-based service to encourage young men to know their status, specifically those with limited access to or are reluctant to attend clinics. Strengthening HIV and/or AIDS education could facilitate better decision-making towards HIV testing among young men.

**Contribution:**

This study contributes to an understanding of young adult men’s knowledge of HIV and/or AIDS and HTS in underserved settings in South Africa.

## Introduction

Globally, millions of individuals have been infected and affected by the human immunodeficiency virus and/or acquired immunodeficiency syndrome (HIV and/or AIDS).^1^ After initially being identified in a homosexual population in the United States of America (USA) in 1981, HIV and/or AIDS has become one of the leading global public health concerns,^2,3^ with more than 75 million people infected since then.^4^ There were an estimated 38.4 million (33.9–43.8 million) people living with HIV at the end of 2021,^5^ of whom 650 000 (510 000–860 000) died from HIV-related causes, and 1.5 million (1.1 million–2.0 million) acquired HIV.^5^

Sub-Saharan Africa (SSA) remains the region with the highest HIV and/or AIDS cases and accounted for 59% of new global HIV infections in 2021.^5^ While South Africa (SA) has the most extensive antiretroviral therapy (ART) rollout programme in the world, it remains the country with the most significant global and continental HIV and/or AIDS epidemic, with more than 8 million people living with HIV and/or AIDS (PLWHA), accounting for 13.7% of the entire population in 2021.^4,6,7,8^ Despite the reduced death rate and the availability of effective treatments, the epidemic remains a concern.^4,8^ In SA, the data from 2019 showed that KwaZulu-Natal (KZN) remained the province with the highest number of HIV and/or AIDS cases, with 27% of the population living with HIV.^9^ The female population bears the brunt of the infection, with a higher infection rate than their male counterparts. A population-based cohort study on age shifts in HIV-1 incidence over a 16-year period (2004−2019) by Akullian et al.,^10^ showed that the incidence rate (per 100 person years [py]) of HIV has declined among young men (15−19 years) by 64% (0.92–0.32) and 25−29 years by 46% (3.91–2.13). In the same study, the HIV incidence rate remained stable among men aged between 30 and 34 years at less than 20% change (3.26–2.78).^10^ Another study by Baisley et al.^11^ among young men aged 20−29 years between 2011 and 2015 found that less than 50% of them tested for HIV in both years, even with the increased coverage of voluntary medical male circumcision (VMMC) offered for free at South African public healthcare facilities. Despite all the targeted HIV prevention efforts,^8^ young people remain at a heightened risk of infection because of risky sexual behaviours, among other reasons.^12^ In an attempt to reduce the spread of HIV and/or AIDS in SA, the National Department of Health (NDoH) has developed various intervention strategies, including a national HIV testing campaign, to increase access and uptake of HIV testing services (HTS), VMMC, pre-exposure prophylaxis (PrEP) and post-exposure prophylaxis (PEP), as well as gender-specific interventions aimed particularly at women.^13,14,15^

Studies have shown that knowledge about HIV and/or AIDS affects attitudes towards testing and preventative practices, with the provision of correct information shown to be essential in lowering the infection rate.^15^ A good understanding of HIV and/or AIDS may also aid in promoting safe and healthy sexual behaviour, although it may not be the only solution to HIV and/or AIDS prevention among young people, particularly adolescents.^16^ While awareness of HIV and/or AIDS is essential, a comprehensive understanding of the pandemic and the factors driving it is also required for effective policy intervention to work.^2^ A study by Tetteh et al.^17^ in SSA found that in the 29 nations that were studied, only 51% of men had excellent knowledge about HIV and/or AIDS, with Rwanda having the highest (76.4%) and Benin the lowest (31.1%). Another study conducted between 2017 and 2018 on young adults (18−24 years) in two high HIV burden districts in SA found that less than 50% of respondents had accurate knowledge about HIV, with many still believing myths about the disease.^6^ The author found that rural young people had low (44.7%) levels of accurate knowledge about HIV prevention in general, although males were slightly more knowledgeable than females (47.3% and 42.1%, respectively) in one of the study districts.^6^ The study concluded that the low knowledge of HIV at the district level was because of the rural setting, low levels of formal education and widespread poverty, which contributed to transactional sex.^6^ However, even when men have the correct knowledge about HIV and/or AIDS, their attitudes and practices are not correspondingly affected, as they continue to participate in risky sexual behaviours.^18,19^ As young people are at heightened risk of acquiring HIV infection because of risky lifestyle choices, it is vital for them to be knowledgeable about HIV prevention methods, as well as encourage them to make consistent use of condoms and consider PEP and PrEP.

A study by Choruma et al.^16^ in Cameroon in 2017, which assessed high school students’ knowledge of HIV and/or AIDS, revealed a high level of knowledge about the disease. This knowledge included modes of transmission, particularly unsafe sexual interactions, and prevention methods. However, when trying to understand sexual behaviour, it is essential to consider other factors, such as peer pressure and power dynamics in romantic relationships, as they influence behaviour. Sources of knowledge of HIV and/or AIDS have been shown to be a critical factor in influencing the behaviour of young people with regard to engaging in safe sexual practices, although it is certainly not the only factor. Although there is increased exposure of the youth to various channels of HIV and/or AIDS information,^16,20^ some may not be correct, with people often not being discerning about their sources of information, which can result in unsafe sexual behaviour.^16^

Choruma et al.^16^ reported that young people were exposed to HIV and/or AIDS knowledge through mass media (television and radio), school teachers, social media, and peers, among others. These findings are consistent with a South African study by Shamu et al.^6^ which found that media utilisation, including mass and social media, influenced HIV knowledge in young people. Media exposure can be effective and influential in educating individuals about HIV and/or AIDS.^21^ However, a study by Fana^4^ in the Eastern Cape noted that school teachers and healthcare workers were the primary sources of HIV and/or AIDS information rather than the mass media. Providing sufficient, accurate, age-appropriate information and health education about HIV and/or AIDS remains central to facilitating better decision-making in sexual choices, which informs young people’s sexual behaviour and practices.^4^

Studies indicate that HIV testing is strongly associated with better HIV knowledge,^22,23^ the common entry point for treatment and care often being HTS, with increased testing efforts being vital to facilitate rapid linkage to these services.^24^ Although personal knowledge and attitudes about HIV are critical, it is essential to recognise that the uptake of HIV testing is influenced by several factors, notably at the individual and community levels, including socio-demographic, economic and behavioural factors.^22^

Studies have consistently found that men engage less than women in health care activities, including HTS, and have worse HIV treatment outcomes.^25^ The provision of factual health education targeting men (as well as women) is key to addressing the low uptake of HTS and necessitates awareness and understanding of the characteristics that enable or disable men to test for HIV and prevent infection.^22^

In addition to factual knowledge, knowing where to test for HIV is key to facilitating access and utilising HTS. A study by Kirakoya-Samadoulougou et al.^22^ in Burkina Faso found that three-quarters of respondents knew where they could test for HIV but did not specify if knowing where to test for HIV translated into actual testing.

With HIV testing being a critical step in preventative efforts and linking those who test positive to care, the low uptake, by men, in particular, indicates the need to explore alternative HIV testing methods, such as HIV self-testing (HIVST). A study by Mhango et al.^26^ in Namibia found low (24%) knowledge and awareness levels of HIVST among the respondents, including men. This finding suggests a need for awareness campaigns if HIVST is to be regarded as an effective strategy to increase testing in hard-to-reach settings, such as young men in rural areas. Such an HIVST strategy might increase the likelihood of achieving HIV prevention and treatment objectives, which, together with community-based HIV testing and counselling services that integrate behaviour modification and HIV prevention communication, could improve uptake of HIVST.^6^ With the above noted, there remains a gap in understanding the knowledge of young men with regard to HIV and/or AIDS and HTS in rural and peri-urban communities in KZN. The aim of this study was, therefore, to explore young men’s knowledge of HIV and/or AIDS, their awareness of HTS in Ladysmith and their willingness to use HIVST.

## Research methods and design

This was a qualitative descriptive research study to better understand and describe young men’s perceptions of HIV and/or AIDS and HTS. The study was conducted in the rural area of Driefontein and peri-urban Steadville near the town of Ladysmith, KZN province. Steadville is a small peri-urban residential township located 4 km outside the town with an estimated population of 25 614,^27^ while Driefontein is 3 km beyond the town limits with a population of 6774.^28^ Young people aged between 18 and 35 years account for 35% of the population of Steadville and Driefontein. Schooling in the area is poor, and there are few job opportunities for young people, with an unemployment rate of over 80%.^29^

The inclusion criteria were those who were aged between 18 and 35 years as this range encompasses the minimum legal age of being an adult, and participants are also old enough to provide informed consent for the study, were willing to participate in the study, did not know the researcher, had a WhatsApp-enabled smart cellular phone and could use the social media application. Previous HTS experience was not required to participate. They needed to be willing to participate in the study and provide informed consent accordingly. For this study, only young men aged between 18 and 30 years participated in the study, which was still in the specified age range. It is important to note that the age category for participation in this study was guided by the South African National Youth Policy (NYP) (2020−2030), which defines young people in South Africa as being between 14 and 35 years old.^30^ Also, the researcher was interested in young men. Human immunodeficiency virus prevention programmes seldom target individuals below 25 years of age, leaving the population aged between 18 and 25 years untargeted for prevention at the programme level.

The researcher used purposive and convenience sampling techniques to select participants for data collection in this study. The researcher called young men in Steadville and Driefontein who had responded to the poster or posters shared on WhatsApp and Facebook communication platforms. The poster contained the researcher’s contact details. Those who were willing to participate sent him a direct message on WhatsApp communication platform expressing their interest, and dates and times for the interviews were scheduled accordingly. Interested young men needed to meet the inclusion criteria and be able to share their personal perceptions of HTS in their personal lives and communities. Eleven participants from the peri-urban Steadville Township were purposively recruited through WhatsApp and Facebook communication platforms.

The researcher applied convenience sampling to recruit participants from the rural Driefontein because he did not receive any feedback from any of the young men through WhatsApp and Facebook communication platforms. In this regard, he sent the poster to an employee from a research institute in Ladysmith, who is actively involved in HIV programmes that young men are part of in that town, and asked him to forward it to his contacts. The researcher was aware of the *Protection of Personal Information Act* (POPIA), No.4 of 2013, which every South African resident needed to abide by from 01 July 2021.^31^ The researcher complied with the POPIA with regard to the re-use of subject data in research.^32,33,34,35^ In this way, participants from Driefontein were found using a list provided by the research institute employee with 13 potential participants who showed interest in participating in it and consented to having identifying particulars and contact details shared with the researcher. The researcher did not ask for the potential participants’ identifying particulars but their willingness to participate. The researcher called them and asked each of them if they were informed about the study and they all agreed. The researcher managed to secure the participation of six participants who met the inclusion criteria. A mutually convenient time for the interviews was agreed upon.

Interested young men needed to meet the inclusion criteria (as stipulated above), be willing to participate in the research study and share their personal views and perceptions of HTS in their communities.

In September 2021, semi-structured interviews (SSI) were conducted via WhatsApp and landline audio calls with 12 young men using an interview guide. The coronavirus disease 2019 (COVID-19) pandemic and the national lockdown prevented researchers from conducting face-to-face interviews. The interviews ranged from 24 min to 1 h and 12 min and were conducted in English and isiZulu, the local language. An additional five young men were interviewed after analysis of the first 12 interviews, after which data saturation was reached. Of the 17 interviews, one was conducted through a landline telephone because of network connectivity. Their informed verbal consent was obtained, and every participant was given an alias to ensure anonymity. All interviews were recorded, transcribed verbatim, translated into English, and thematically analysed.

### Ensuring trustworthiness

To ensure the credibility of the study findings, the researcher relied on the information provided by the participants if they were the right people by reiterating their names, surname, and location. The researcher audio recorded all the interviews with permission from the participants, to ensure the credibility of the findings. He also ensured that the participants were informed about the study’s purpose, and took them through the entire informed consent process. Additionally, he contacted his supervisor for a debriefing session, reflected on the data collection process, and reached out to his colleagues for advice on the best way to analyse the collected data.

### Ethical considerations

Ethical clearance to conduct this study was obtained from the University of KwaZulu-Natal Humanities and Social Sciences Research Ethics Committee (No. [HSSREC/00000588/2019]) and the KwaZulu-Natal Provincial Health Research and Ethics Committee of the Department of Health (No. KZ_202008_006).

## Results

Seventeen young men participated in the study, all of whom were African (black people), IsiZulu language native speakers and single. Eleven were from the peri-urban Steadville township, while six were from rural Driefontein. The two oldest respondents were 30 years old, while the youngest one was 18. Most were employed (6), full-time high school learners (3), higher education, tertiary, students (3), unemployed (3), and self-employed (3). One of the high school learners was also self-employed. Additionally, most respondents were either busy with or had completed Grade 12 and above. Two respondents were, in Grade 11 and one was in Grade 10. It is important to note that while some participants mentioned that they were sexually active, others did not indicate if they were sexually active although they still tested for HIV. Only one participant identified as gay. Themes and sub-themes are indicated in Table 1.

**TABLE 1:** Themes and sub-themes.

Theme	Sub-themes
1. Knowledge of HIV and/or AIDS	Understanding of HIV and/or AIDS
	Knowledge of HIV and/or AIDS facilitates a better understanding
2. Knowledge of HIV testing services	-
3. Knowledge of HIV self-testing	Awareness of HIVST
	Advantages and disadvantages of HIVST
4. Sources of HIV and/or AIDS and HTS information	Knowledge of HIV and/or AIDS sources
	Knowledge of HTS sources
5. Knowledge of HTS testing sites	Places where HTS can be accessed and utilised

HIV, human immunodeficiency virus; AIDS, acquired immunodeficiency syndrome; HTS, human immunodeficiency virus testing services; HIVST, human immunodeficiency virus self-testing.

### Theme 1: Knowledge of HIV and/or AIDS

Knowledge about HIV and/or AIDS determines the extent to which individuals comprehend HIV in its entirety and influences their decisions about testing for HIV and participating in safe sexual practices. This theme related to how the men described HIV and the extent to which their knowledge facilitated their understanding of the disease.

#### Sub-theme 1: Understanding of HIV and/or AIDS

These research findings revealed that most respondents perceived HIV and/or AIDS as interchangeable terms. Their understanding of HIV is that it is a disease that is neither deadly nor curable, but it is manageable with treatment adherence to avoid reaching the advanced stage where the body resists medication and possibly leads to death. Taking one pill a day was understood as acceptable and doable. In this regard, respondents were optimistic and had the following to say:

‘… HIV and/or AIDS is a disease that exists in our society, it is not curable, but it is preventable and if it happens that you are infected with the virus, there are medications that you can use not to cure it but to maintain it within your system and live your life like any other person who does not have HIV and AIDS.’ (Participant 8, 22 years old, male)

Although most respondents understood HIV and/or AIDS as a disease, one participant understood HIV and/or AIDS as being separate from one another, as indicated by his description:

‘I can say it a bit different, because HIV can be treated and managed when caught early, and you can live for 30 years and above. Once it is AIDS, then it becomes difficult to treat because it has then destroyed a lot of your body cells.’ (Participant 14, 25 years old, male)

Human immunodeficiency virus and/or acquired immunodeficiency syndrome was described by some respondents by means of its mode of transmission, mainly through unsafe sexual intercourse and blood, as Participant 16 noted:

‘I can say it [*HIV and/or AIDS*] is a sexually transmitted disease, of which you can get in different ways, having unprotected sex or touching blood samples of someone who has HIV … when you are not wearing protection, like gloves.’ (Participant 16, 26 years old, male)

#### Sub-theme 2: Knowledge of HIV and/or AIDS facilitates a better understanding

When asked if their knowledge of HIV and/or AIDS changed from the time they knew nothing about it to the time when they understood it, the respondents acknowledged that increased knowledge because of exposure to information at school during life orientation lessons, clinics, social media, and the internet helped them to understand HIV and/or AIDS better. The respondents reported the following:

‘What I can say is that, from that time I did not know much, I thought maybe HIV was a disease that infects only a certain group of people, but as I got information along the way, I learnt that anyone could get HIV.’ (Participant 16, 26 years old, male)

‘… I used to think that if you have HIV, you think about it every day that you have it, I had a negative attitude about HIV. I do understand it clearly and better now because now, I know what it is and how to treat it.’ (Participant 17, 18 years old, male)

One respondent highlighted the role that stigma played in his lack of understanding of HIV and/or AIDS and that more information about HIV and/or AIDS reduced the stigma:

‘We grew up when there was a stigma about it, but (I) learned that HIV is something that you could live with … stigma from the community, people will treat them differently and talk badly about someone who had it. At that time, because I was growing up, I was part of the community, so I thought the same … but when I got more knowledge as I studied further, I realized that I was getting worked up over nothing … like you had this mentality that you must be scared of an infected person. But as time went on I realised that this is not something that you just contract, and there are things that you need to do in order to contract it, so there’s no need to be scared of an infected person.’ (Participant_3, 20 years old, male)

### Theme 2: Knowledge of HIV testing services

Apart from the knowledge of HIV and/or AIDS, respondents were aware of HTS, with the findings showing that although they had all utilised HTS and were aware of such a service, there were still gaps in their knowledge. Participant 11 stated: ‘I am not familiar with it (HIV testing). I think it’s the amount of knowledge we have about it is not enough’. (Participant 11, 28 years old, male). Another participant stated: ‘I have a very basic knowledge around HIV testing …’ (Participant 1, 30 years old, male)

One respondent mentioned that although he was aware of HTS and continues to use the service, he still had only basic knowledge of HTS.

‘I am aware of HIV testing services, and I’ve done it so many times because as a person living in this world, I have been sick here and there … I would not say I know a lot about HIV testing services, because most people know what they need to know because they would be going through some stuff, so they only know things when they are going through stuff. So, I wouldn’t say I know a lot about HIV testing services.’ (Participant 2, 30 years old, male).

### Theme 3: Knowledge of HIV self-testing

This theme related to understanding HIVST, with two sub-themes emerging on the awareness and the advantages and disadvantages of HIVST.

#### Sub-theme 1: Awareness of HIV self-testing

The findings revealed that most respondents were unaware of or knowledgeable about HIVST as an alternative method of HIV testing, with one respondent being aware but not having used it. Participant 14 said: ‘I have not heard about it (HIVST)’ (Participant 14, 25 years old, male). Participant 10 also stated: ‘I am aware of HIVST, but I have never used it’. (Participant 10, 26 years old, male)

#### Sub-theme 2: Advantages and disadvantages of HIV self-testing

The researcher briefly explained HIVST to those unaware of what it was. The respondents noted the advantages and disadvantages of HIVST, particularly in the absence of counselling and not knowing where to get treatment should people test positive for HIV:

‘I would faint if I was to test myself. There’s that counselling that you get before the results come out … it is important and when you test yourself you don’t get it [*counselling*] … it is needed … as scary as it is … but I think it would be advisable to go to the clinic and get professional help to handle the testing correctly, and if one does it himself, he won’t handle it well and won’t go to get help at the clinic’ (Participant 3, 20 years old, male)

‘I do not think that it [*HIV self-testing*] is a good thing because I will need someone to counsel me first, especially if I test positive for HIV.’ (Participant 11, 28 years old, male)

Some respondents viewed HIVST as a possible alternative for, young, men who fear facility-based HTS but expressed concerns with the approach:

‘I would use it [*HIVST*]. But once tested, where would I go for treatment? Because when I am at home, I will not be able to get treatment. Firstly, at home, you do not have advisors [*counsellors*], you are alone in your room testing yourself. If there are problems … you first get advice at the clinic, get tested then while waiting for your results, you talk to the advisor about living with HIV. You see, it is better to go to the clinic that to test yourself at home.’ (Participant 17, 18 years old, male)

‘… as people, we are afraid of what we might find when we go to clinics, because sometimes nurses, when you are positive, they have to take you for counselling and advise you, some of us do not want that, so self-testing is the way to go for such people. It might help a lot of people. There are guys I have met who are not keen on going to the clinic, but one of them brought these kits and they were willing to use them and test themselves at home, alone. So, I think that is how it makes it easier than going to the clinic.’ (Participant 14, 25 years old, male)

### Theme 4: Sources of HIV and/or AIDS and HIV testing services information

This theme related to HIV and/or AIDS and HTS information sources, where various channels were reported.

#### Sub-theme 1: Knowledge of HIV and/or AIDS sources

Information about HIV and/or AIDS was found through various channels, including the media (internet, news, tabloids, and social media), educational institutions and health facilities (clinics and hospitals). Most stated that school and media platforms were their primary sources of HIV and HTS knowledge. One particiapnt stated: ‘I get mine from the news, newspaper, social media and the internet.’ (Participant 11, 28 years old, male). Another participant stated said: ‘I get mine from school and the internet’ (Participant 6, 21 years old, male). Participant 5 inidcated: ‘I got my knowledge at school from educators’ (Participant 5, 29 years old, male).

#### Sub-theme 2: Knowledge of HIV testing services sources

Young men obtained knowledge of HTS from various channels, which helps to release some pressure of not going to the clinic to get more information. They expressed the following:

‘I listen to the radio a lot, I hear them talking about knowing your status, going to the clinic and getting tested so if there is anything amiss, you can act immediately, so I went to get tested so I can move on with my life knowing what is happening. I get a lot of information from Ukhozi FM.’ (Participant 14, 25 years old, male)

‘I usually find it [*HTS information*] on google, I am very familiar with it. I use google to find factual information.’ (Participant 13, 21 years old, male)

Health facilities, especially clinics, were sometimes young men’s knowledge source of HTS, and Participant 12 expressed the following:

‘I get my HIV testing knowledge at the clinic because after testing they tell you to come back after a particular period, you see all of that. They tell you to come back after a certain period of time to test, and they also encourage us to bring our partner for HIV testing as well and not go alone.’ (Participant 12, 25 years old, male)

### Theme 5: Knowledge of HIV testing services testing locations

This theme related to young men’s knowledge of where people can test for HIV.

#### Sub-theme 1: Places where HIV testing services can be accessed and utilised

Participants knew that HTS is available at some non-governmental organisations and health facilities, including clinics and hospitals. More than half of the respondents identified health facilities, mainly clinics and hospitals, including mobile clinics, as the main sites where people can test for HIV. Mobile clinics were cited as necessary as they reduce the distance people travel to the health facility, particularly in rural areas, where fixed clinics can be far from where people live. In this regard, respondents said the following:

‘Clinic, hospital, and there are also vehicles going around here in the rural areas that test for free, you get into the car, get tested and leave.’ (Participant 17, 18 years old, male)

‘In my area, there’s a microbus that goes around and my brother told me that if you want to test you can stop it and they test you on the spot … I’m not sure how true that is … and the clinic because it is always available.’ (Participant 9, 29 years old, male)

Apart from health facilities, some participants were aware that there are public health non-governmental organisations (NGOs) where people can test for HIV. Participant 10 said:

‘You can test at the clinic, mobile clinics are also available, there are many places that test, including pharmacies, where you can go if you don’t like to test at the clinic, there are NGOs that put up tents and test.’ (Participant 10, 26 years old, male)

One participant identified health facilities as places where people can test for HIV:

‘I think the clinic is always open and available. It’s better now because there are cars, those white 22-seater cars. They drive around the streets, and I don’t think a day goes by without seeing one. They always drive around the township, and I think they also have their own daily testing targets.’ (Participant 5, 29 years old, male)

Participant_5 further suggested that schools could be targeted as a place to test learners. However, he acknowledged the difficulty in carrying out this exercise in schools and suggested that learners be tested separately:

‘… especially schools to test them and before they test them, they need to counsel them first. There could be days where they visit schools. Maybe they can counsel them today and start testing them today if they are able to counsel everyone on the day, but they should not disrupt teaching and learning time. I think it would be something of that nature … I think if they can counsel every learner separately and test because for others, it would be difficult when they are in the presence of others.’ (Participant 5, 29 years old, male)

One respondent revealed that, in addition to clinics and hospitals, people could test for HIV upon blood donation:

‘People can test for HIV at the clinic and hospital … oh and the mobile clinics that come to our college and travel across the country, as well as those who conduct blood donation, because they test for HIV before drawing blood’ (Participant 7, 22 years old, male)

This section provided the description, analysis, and interpretation of the study findings from young men, which showed their perceptions of the knowledge of HIV and/or AIDS, HTS, HIVST, sources of HIV and/or AIDS and HTS information, as well as their knowledge of places where young men and other people can get tested for HIV, including public healthcare facilities, public health organisations, schools, and blood donation centres.

## Discussion

The research findings revealed that young men in the rural Driefontein and peri-urban Steadville Township had fairly good knowledge about HIV and/or AIDS, including modes of transmission, and how such knowledge influences their decision to test for HIV. Nationally, as a rule of thumb, the following HIV services are provided at the Department of Health (DoH) primary health care facilities: HIV prevention (PrEP, PEP, VMMC, HTS, etc.), referral and linkage to HIV treatment and care. For most participants, both HIV and AIDS were regarded as the same disease and not separate from one another. This is different from the finding by Ryan et al.^36^ in 2020 among male and female patients in an emergency department. In this study, Ryan et al.^36^ assessed whether HIV knowledge and attitudes influenced HIV testing acceptance and found that men and women are aware that HIV causes AIDS and could highlight the difference between them. Young men need to understand the difference between HIV and AIDS to be knowledgeable about the various phases of the disease progression.

There was a common understanding that HIV and/or AIDS is not curable, regardless of the medical interventions, but is treatable and that medication adherence is essential upon testing positive for HIV to avoid reaching the stage of AIDS, where it becomes a challenge to contain the disease, which is potentially life-threatening and may result in death. This understanding is essential, as early detection of the virus enables persons who have tested positive for HIV to seek medical help and continue to live a normal life if they adhere to ART.^37,38,39^ Knowledge of HIV and/or AIDS was further understood by means of the various modes of transmission, including unsafe sex with a person living with HIV and by touching their blood without wearing protective equipment. These findings are consistent with that of Nubed et al.^15^ and Aloni et al.,^38^ who reported that participants were aware of various HIV and/or AIDS modes of transmission. However, their findings did not indicate if participants had any knowledge of issues such as HIV viral load suppression, which is acquired through treatment adherence and reduces the possibility of infection. There is a need for further research to gauge young men’s understanding of HIV viral suppression and if this impacts their sexual behaviours, especially the use of condoms and when they are in serodiscordant relationships.

Although the young men in this study had some knowledge of HIV and/or AIDS, including modes of transmission and that HIV is manageable, the information was not comprehensive. This is consistent with the study by Tetteh et al.,^17^ who assessed the level of awareness of HIV and/or AIDS knowledge and HIV testing in 29 countries in SSA. Being equipped with comprehensive and accurate knowledge about HIV and/or AIDS and HTS will put young men in a better position to make informed decisions about whether or not to test for HIV, should the need arise.^39,40,41^

Although the findings indicate that the respondents knew about HIV and AIDS and were aware of HTS, they stated they had limited knowledge of HTS. This contradicts the findings of Chimoyi et al.,^41^ who noted that being aware of HIV testing and having better knowledge about what services are available increase the likelihood of getting tested. In this regard, knowing about HIV and/or AIDS does not always translate to adequate knowledge and awareness of HTS. Respondents did not mention where the knowledge gap is, which warrants further investigation. Furthermore, based on the findings, the limited HTS knowledge did not translate into low access and utilisation of HTS, as all young men in this study had had a recent HTS experience.

HIV self-testing has been reported to be an alternate to clinic-based HTS,^42,43^ potentially diminishing barriers to HTS that individuals encounter at clinics.^44^ Most of the young men were neither knowledgeable nor aware of the existence of the HIVST approach.^45^ After having the approach briefly explained to them, most respondents expressed disapproval of it, which contradicts the findings by Hamilton et al.,^46^ who noted its acceptability among men in SSA. The main concern about HIVST was the absence of counselling that is provided at the health facilities (both fixed and mobile) that offer HTS.^47,48^ The young men felt that they would not be able to cope emotionally with the possibility of a positive HIV result in the comfort of their homes without having received pretest counselling. Although some men remarked that they would use the HIVST kit, they also considered the element of counselling to be key to treatment initiation and the journey to acceptance, which is available in health facilities. This is in line with the findings by Bwalya et al.,^44^ which revealed the need for supervised HIVST for first-time testers, where they could be educated about and shown how to use the HIVST kit. This needs further exploration, as men (particularly young men) are a hard-to-reach group, and HIVST would provide testing options for those who were unable to or did not want to visit healthcare facilities for HTS. Although previous studies have indicated a perceived lack of the kit’s accuracy,^44,49^ which was not the case in this research study, the participants did not see HIVST as a suitable way to extend testing services.

In this study, young men indicated that their source of knowledge of HIV and/or AIDS and HTS included media platforms, schools, and clinics. This is consistent with the findings by Oppong Asante^50^ and Ndlovu,^47^ who noted that respondents were aware of the knowledge sources of HIV and/or AIDS and HTS. Although the young men reported multiple channels of knowledge sources, the majority indicated that the school curriculum and media platforms, such as the internet and social media, were the main sources of information on HIV and/or AIDS and HTS. They were, however, unaware of the shortcomings of their information sources, especially the school curriculum and social media. The main reasons that the internet emerged as a key source of HIV and/or AIDS knowledge may be that it is widely available, and is used extensively by young people, which can be accessed through their cellphones.^40,51^ A 2019 study by Chinoza^52^ among university students on mobile internet access and affordability among youth in South Africa further confirmed the extensive utilisation of mobile internet access. However, there is a need for research on the number of youths who have access to the internet in South Africa. Media exposure has been found to increase HIV testing among young men and women in SSA over the last 10 years, with females experiencing a greater increase than males.^51^ Overall, young men knew where to get information, but the findings did not reveal how much of the information was sourced from reputable sources. This is an important consideration, as many myths and untruths are found on the internet and learning how to discriminate good information from bad is an essential skill that is not taught.

The school curriculum was an important source of information about HTS, and the findings from this research suggest that much of the factual content regarding HIV and/or AIDS in the South African educational curriculum, especially during Life Orientation, is appropriate.^53^ This finding is consistent with those from a study on knowledge, attitudes and practices regarding HIV and/or AIDS among high school secondary learners in Fako Division in Cameroon by Nubed et al.,^15^ which revealed that sex education in school was the commonest source of HIV and/or AIDS knowledge. However, it is unclear how comprehensive the curriculum is on HIV and/or AIDS as there were gaps in their knowledge, specifically about HTS. There is also a need to explore and understand factors contributing to sexual behaviour among high school learners and how their attitudes and practices can be changed. Schools were also mentioned as a potential environment to conduct HIV testing by targeting learners, being informed by the possibility of some learners being sexually active or living with HIV but not being aware of their status. Although some respondents had tested for HIV at school, there was concern among them because of confidentiality issues. There is a need to explore HTS conducted in schools with learners and educators, the challenges this may pose, and to develop innovative school interventions to facilitate HTS and behaviour change among learners. The focus on HIV and/or AIDS and HIV testing in the education field through appropriate effective strategies has greater potential to improve HIV and/or AIDS prevention than other interventions.^18,54^ Improvements in HIV and/or AIDS prevention programmes in the school context could potentially be achieved by emphasising the importance and benefits of HIV testing and practising safe sex. In addition, educators need to be adequately capacitated about HIV and/or AIDS and HTS to avoid passing on non-factual and insufficient information to learners.^55^

All respondents were aware of places for HIV testing, including public healthcare facilities, such as hospitals and clinics, and mobile clinics. This is consistent with the findings of Abiodun et al.^40^ and Oppong Asante,^50^ who reported that young men were well aware of places in their communities and town where they could test. Although young men knew about the presence of mobile clinics in their surroundings, none of the respondents mentioned ever having made use of their services for testing. The exception was when a respondent went to the clinic to test for HIV and was referred to the mobile bus parked inside the premises. The availability of mobile buses at healthcare facilities may improve the uptake of HTS among young men who are reluctant to test for HIV in the allocated HTS consulting rooms inside the facility. This finding is consistent with a previous study, which reported that mobile clinics in communities facilitate improved HIV testing in men.^56^ Further inquiry is needed to understand why young men seldom use mobile clinic HTS services around their communities.

Moreover, while mobile clinics reduce the distance to health services and increase access to HTS, none of the respondents cited distance as an impediment in their journey and experience with HTS. It can be argued that distance was not a factor of consideration for young men in both communities. One respondent mentioned a blood donation centre as one of the places to test for HIV, but he did not mention if he has utilised the centre to donate blood or to test for HIV. In this regard, blood donation centres might be used to test for HIV, especially if confidentiality is guaranteed, as the literature has noted a lack of confidentiality by healthcare providers (HCPs) in public healthcare facilities as a concern. However, the blood transfusion services make it clear in their assessment forms that they are not HIV testing centres and no one should donate blood to determine their HIV status. An HIV test is only done after the blood has been taken, with no reports being given back to those who provide regarding any disease or other condition found in their blood. There is a need for a quantitative inquiry to investigate the relationship between socio-demographic information, HIV and/or AIDS knowledge, and the likelihood of testing for HIV in underserved settings.

### Study limitations

Given the qualitative nature of the study, these findings cannot be generalised to other populations. In addition, these findings do not represent the experiences of all young men in Steadville and Driefontein communities. Because of the use of WhatsApp and landline telephone audio calls as a data collection tool, the researcher could not derive any interpretation and meaning from the respondents’ facial and body language and probe further based on these observations. However, the study provided valuable insights into young men’s HTS, highlighting areas for further research in hard-to-reach settings.

One of the challenges of using WhatsApp communication platform was that there were internet connectivity issues. Another one was that some participants did not have data to take a call. To mitigate this, participants were reimbursed with R29.00 one day data that varied between 300 MB and 1 GB, depending on the participants’ network provider. Those who had used their own data were compensated with replacement data of the same value.

It is also worth noting that another challenge was the recruitment of participants, whereby the researcher resorted to convenience sampling instead of purposive sampling to recruit Driefontein participants because of a lack of response from them. Also, the accessible population was limited to the researcher and the employee from a research institute in Ladysmith.

## Conclusion

While the young men were fairly knowledgeable about HIV and/or AIDS and HTS testing and knew places where people can test for HIV, there were gaps in their knowledge of HTS (most young men possessing basic knowledge of HTS) and HIVST (the majority of young men were unaware about HIVST) that could affect their uptake of HTS and influence their sexual behaviour, putting them at risk of infection. Human immunodeficiency virus and/or acquired immunodeficiency syndrome and HTS educational and awareness campaigns through various mediums need to be conducted to highlight the importance and benefits of HIV and/or AIDS and HTS. HIV self-testing should be explored as an alternative mechanism to encourage young men to know their status for their second round of testing once the counselling has occurred at their initial test. However, HIV testing should be encouraged among young men to enable them to lessen the anxiety of not being aware of their HIV status.
